# Association of serum 25‐hydroxy vitamin D with obesity‐related indices in Chinese adults: A cross‐sectional study

**DOI:** 10.1002/fsn3.2201

**Published:** 2021-02-26

**Authors:** Zhongxia Ren, Ai Zhao, Yan Wang, Liping Meng, Ignatius Man‐Yau Szeto, Chenlu Yang, Meichen Wang, Jian Zhang, Wei Wu, Peiyu Wang, Yumei Zhang

**Affiliations:** ^1^ Department of Nutrition and Food Hygiene Peking University Health Science Center Beijing China; ^2^ Vanke School of Public Health Tsinghua University Beijing China; ^3^ Inner Mongolia Dairy Technology Research Institute Co. Ltd. Hohhot China; ^4^ Yili Innovation Center Inner Mongolia Yili Industrial Group Co. Ltd. Hohhot China; ^5^ Department of Social Science and Health Education Peking University Health Science Center Beijing China

**Keywords:** anthropometry, obesity, vitamin D

## Abstract

Obesity has been a well‐known risk factor of low‐level serum vitamin D. Compared with the traditional obesity‐related indicator (body mass index, BMI), associations for two novel anthropometric indices, a body shape index (ABSI) and body roundness index (BRI) with vitamin D deficiency or insufficiency, still remain unclear. This study aimed to assess the associations of serum 25‐hydroxy vitamin D (25(OH)D) status with these three obesity‐related indices among Chinese adults. A total of 1666 individuals were included. Anthropometric measurements were performed to calculate the indices, and fasting blood was collected to determine serum 25(OH)D deficiency (<12 ng/ml) and insufficiency (12–20 ng/ml). Deficiency or insufficiency of 25(OH)D was found in 37.5% and 43.1% of the participants, respectively. After adjustment for potential confounders, a significantly increased prevalence of 25(OH)D deficiency was observed for higher ABSI (OR _Q4 vs Q1_ 2.334, 95% CI 1.458–3.734; *p*_trend_ = 0.002) and BRI (OR _Q4 vs Q1_ 2. 215, 95%CI 1.365–3.594; *p*_trend_ = 0.010), and for higher BMI in men. Regarding 25(OH)D insufficiency, a significant association was also found for ABSI (OR _Q4 vs Q1_ 2.372, 95%CI 1.558–3.612; *p*_trend_ = 0.001). The area under the ROC of ABSI (0.731, 95%CI 0.687–0.774) for predicting a low level of 25(OH)D was significantly larger than that of BMI (0.695, 95%CI 0.649–0.741) in men, but not in women. A positive association between obesity and lower 25(OH)D serum concentration was found among Chinese adults. Besides BMI, novel obesity‐related indicator, ABSI and BRI were associated with lower serum 25(OH)D to some extent, and further studies are needed to clarify their potential to be used as screening tools in clinical practice.

## Graphical abstract

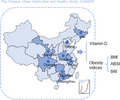

## 1 INTRODUCTION

Vitamin D, as a nutrient closely related to human health, has been studied for a long time. In addition to its well‐established effects on the musculoskeletal system (Bischoff‐Ferrari, 2012; Holick, 2005), the relationship between vitamin D and obesity has drawn more and more attention (Pereira‐Santos et al., 2015; Pourshahidi, 2015). Considering that vitamin D deficiency has emerged as one of the major global public health problems concomitantly with obesity, it is important to elucidate the underlying mechanism and implement early interventions (Holick & Chen, 2008; James, 2018; Pereira‐Santos et al., 2015).

Vitamin D3 or D2 is mainly synthesized in the skin in response to solar ultraviolet B (UVB) radiation and in lesser amounts from dietary or supplementary intake (Holick et al., 2011). As a fat‐soluble vitamin, it tends to be stored in and released from adipocytes into the circulation, transported to the liver, and then converted to 25‐hydroxy vitamin D (25(OH)D), the latter form being recognized as a general indicator of vitamin D status (Holick, 2007). According to previous studies, possible hypotheses underlying vitamin D deficiency occurring in obese subjects include excessive sequestration in adipose tissue, less sun exposure due to a sedentary/indoor lifestyle, decreased expression of vitamin D‐dependent receptors, changes in the gut microbiota, gut‐derived metabolites, simple volumetric dilution, and so on (Barrea et al., 2019; Cheng et al., 2010; Drincic et al., 2012; Looker, 2007; Malmberg et al., 2014; Martini & Wood, 2006).

Growing studies revealed that it is reasonable to predict vitamin D deficiency using obesity‐related anthropometric indices, which has the advantage of being simple, inexpensive, and noninvasive. Body mass index (BMI), as a traditional anthropometric index according to height and weight, is possibly the most commonly assessed indicator of obesity in relevant studies (Wood et al., 2012). Systematic reviews and meta‐analyses have revealed a significant correlation between serum 25(OH)D and BMI, albeit with gender and country development status‐related heterogeneity (S. Rafiq & Jeppesen, 2018; Saneei et al., 2013). Despite its widespread use, it should be emphasized that the capacity of BMI to distinguish fat from muscle or to reflect fat distribution is limited (Aly et al., 2016). Further, a recent study indicated that different body fat distributions may make special contributions to 25(OH)D concentrations, as visceral adipose tissue (VAT) showed a closer correlation than abdominal subcutaneous adipose tissue (aSAT) or total body fat (TBF) (R. Rafiq et al., 2019).

Two novel anthropometric indices, a body shape index (ABSI) and body roundness index (BRI) which were first published in 2012 and 2013, took body shape into account. The ABSI was calculated by standardizing waist circumference (WC) for height and BMI. Its inventors, Krakauer NY and Krakauer JC, have claimed that ABSI appeared to be significantly related to abdominal adipose tissue (AAT), and superior to BMI or WC for predicting premature death (Krakauer & Krakauer, 2012). The BRI, based on height and WC, was developed by Thomas DM et al. and considered an improved predictor of body fat and VAT (Thomas et al., 2013). To date, ABSI and BRI have been associated with a series of chronic diseases (Ji et al., 2018), while, to the best of our knowledge, there are only two studies relevant to 25(OH)D status. Both of the studies reported a positive association between the obesity‐related indices and lower vitamin D levels; however, further studies are still needed since their study sample only represented older Portuguese or southern Chinese adults, respectively (Sousa‐Santos et al., 2018; Zhu et al., 2019).

Thus, the aim of the present study is to evaluate the association between obesity‐related indices (ABSI, BMI and BRI) and serum 25(OH)D status and to examine the performance of these two new and one old indices of screening for low‐level 25(OH)D risk in Chinese adults.

## 2 MATERIALS AND METHODS

### 2.1 Subjects

The data in this study were from the Chinese Urban Adults Diet and Health Study (CUADHS), which was a cross‐sectional study conducted from March to July 2016. As described in detail previously (Zhao et al., 2017), a multistage sampling method was adopted for participant recruitment. First, eight cities, including two first‐tier cities (Beijing, Guangzhou) and six nonfirst‐tier cities (Chengdu, Chenzhou, Jilin, Lanzhou, Wuhu and Xuchang), were purposively selected based on differences in geographical location and economic status. Second, a convenience sampling method was applied to select two communities in each first‐tier city or one in nonfirst‐tier cities, respectively. According to resident registration forms, potential participants were then randomly recruited via telephone by local trained health workers. The inclusion criteria were local residents or those who had lived in an urban area for more than one year. The exclusion criteria were subjects with disabilities, infectious or mental diseases, memory problems, or pregnant and lactating women.

The sample size was calculated based on a formula for cross‐sectional study:

(1) $$n=\frac{Z_{\alpha /2}^{2}\;p\left(1‐p\right)}{d^{2}}$$

where n was sample size required, α was significant level set as 0.05, *p* was estimated prevalence of vitamin D sufficiency (≥20 ng/ml) set as 0.3 (Lu et al., 2009), *q* was 1‐*p*, and *d* was the permissible error set as 0.1*p*. A minimum sample size of around 900 was calculated as the number needed to satisfy the requirements. Finally, a total of 1806 individuals were invited and 1739 were enrolled. The current analysis consisted of 1666 individuals as 43 were excluded due to the lack of a blood sample and a further 30 participants were excluded due to missing values of key variables (anthropometric information; education level; household monthly income).

### 2.2 Sociodemographic characteristics and lifestyle factors

Data on sociodemographic characteristics (e.g., age, sex, education level, and household monthly income) and lifestyle factors (current smoking status, drinking status, physical activity over the last seven days, and use of vitamin D supplements) were collected by trained interviewers using a uniform questionnaire. Based on the International Physical Activity Questionnaire (short version) (IPAQ group, 2005) used in the questionnaire, a continuous score expressed as metabolic equivalents of tasks (METs) was calculated and then transformed into tertiles, from the lowest to the highest and marked as the low, medium, and high groups of physical activity. According to the latitude, eight cities were divided into two groups: lower latitude (Chengdu, Chenzhou, Guangzhou, Lanzhou, Wuhu, and Xuchang) and higher latitude (Beijing and Jilin) than 37°.

### 2.3 Food consumption

Daily consumption of several food items in one recent month was calculated using a semiquantitative Food Frequency Questionnaire (semi‐FFQ), including cereals and potatoes, vegetables, fruits, meat and poultry, aquatic products, eggs, milk and dairy products, and soybeans and nuts. Standard reference picture books, bowls, plates, and spoons were used to assist the estimation of dietary intakes. To include food consumption into the multivariate analysis, we converted the continuous form of food consumption to a binary variable (deficiency or not), according to the Dietary Guidelines for Chinese Residents (version 2016) (Chinese Nutrition Association, 2016).

### 2.4 Obesity‐related indices

Height (m), weight (kg), and waist circumference (WC, m) were recorded by trained researchers or professional nurses using a standardized procedure and identical equipment. BMI, ABSI, and BRI were calculated according to the following formulas:

(2) $$\mathrm{BMI}=\frac{Weight\;}{{\mathrm{Height}}^{2}}$$

(3) $$\mathrm{BRI}=364.2‐365.5\times \sqrt{1‐(\frac{{\left(WC/(2\pi )\right)}^{2}}{{\left(0.5\times Height\right)}^{2}})}$$

(4) $$\mathrm{ABSI}=\frac{\mathrm{WC}}{{\mathrm{BMI}}^{2/3}\times {\mathrm{Height}}^{1/2}}$$

According to the China overweight/obesity medical nutrition expert consensus (version 2016) (Drafting committee of Chinese consensus on overweight/obesity medical nutrition therapy, 2016), underweight, normal weight, overweight, and obesity were defined as a BMI < 18.5, 18.5–23.9, 24–27.9, and ≥ 28 kg/m^2^, respectively.

### 2.5 Serum 25(OH)D concentration

Fasting venous blood samples were drawn in the morning to assess serum 25(OH)D concentrations (25(OH)D2 and 25(OH)D3). All blood samples were transported to Beijing by cold chain and tested in a qualified laboratory (Lawke Health Laboratory, Beijing, China). Serum 25(OH)D was detected by liquid chromatography–tandem mass spectrometry (LC‐MS/MS), with lower limits of quantification of 2 and 1 ng/ml, via a high‐performance liquid chromatograph (Agilent 1,100; Agilent Technologies Inc., Santa Clara, CA, USA) and a mass spectrometer (API4000Q trap; AB SCIEX LLC., Redwood City, CA, USA).

The deficiency, insufficiency, and sufficiency of 25(OH)D were identified according to the Application Guideline for Vitamin D and Bone Health in Adult Chinese (2014 Standard Edition) (Liao et al., 2014), with a concentration of < 12, 12–20 and ≥ 20 ng/ml, respectively.

### 2.6 Statistical analysis

IBM SPSS version 22.0 (International Business Machines Corporation, Armonk, NY, USA) and R 3.6.3 were used for statistical analysis. Categorical variables were described as proportions, and continuous BMI, ABSI, and BRI were presented as median (interquartile range, IQR) after normality tests. Chi‐squared analysis or Kruskal–Wallis tests were used for single factor analysis across different 25(OH)D status. Quartiles of ABSI and BRI were calculated by sex and marked as Q1–Q4 from the lowest to the highest.

Multinomial logistic regressions were carried out to analyze the relationship between BMI, ABSI or BRI, and serum 25(OH)D deficiency or insufficiency, with the sufficiency group as the reference. The following confounders with a *p* ≤.10 of single factor analysis were included in the adjusted model: sex, age, education level, monthly household income, dietary intakes (vegetables, fruits, meat and poultry, aquatic products, and use of vitamin D supplements), current smoking and drinking status, physical activity, and latitude of survey points. Combing 25(OH)D deficiency and insufficiency into one group, multivariate binary logistic regressions were applied to calculate the predicted values. The values were then used to establish receiver operating characteristic (ROC) curves and areas under the curves (AUCs) to estimate the prediction capability of three indices for low‐level serum 25(OH)D (<20 ng/ml). Further, the Delong test was used to compare the AUCs. Multivariate analyses mentioned above were also stratified by sex. In addition, to account for missing values, multiple imputation was performed using the mice package (Buuren & Groothuis‐Oudshoorn, 2011) in R software. Sensitivity analyses were conducted in which the main analyses were repeated with imputed data. The level of statistical significance in this study was set at 0.05.

## 3 RESULTS

### 3.1 Characteristics and 25(OH)D status of the study sample

A total of 1666 participants (34% men) were included in this study. The average age was 50.3 ± 17.4 years. Overall, the median level of serum 25(OH)D was 14.1 ng/ml (IQR 8.3). The percentage of adults with 25(OH)D deficiency or insufficiency was 37.5% (24.7% in men and 44.1% in women) and 43.1% (48.2% in men and 40.5% in women), respectively. However, only 8.5% reported intake of vitamin D supplements.

Descriptive data are presented in Table 1. Among all the studied variables, sex, age, education level, monthly household income, dietary intakes (fruits, meat and poultry, aquatic products, and use of vitamin D supplements), current smoking and drinking status, physical activity, and the latitude of survey points were significantly relevant to 25(OH)D status (*p* <.05), as well as ABSI.

**TABLE 1:** Characteristics of the study sample by 25(OH)D status, *n* (%) or median (IQR)

Variables	25(OH)D			*p* value
	Deficiency (*n* = 625)	Insufficiency (*n* = 718)	Sufficiency (*n* = 323)	
Sex				
Men	140 (22.4)	273 (38.0)	153 (47.4)	**<.001**
Women	485 (77.6)	445 (62.0)	170 (52.6)	
Age (years)				
18 ~ 44	303 (48.5)	239 (33.3)	78 (24.1)	**<.001**
45 ~ 64	191 (30.6)	249 (34.7)	120 (37.2)	
≥65	131 (21.0)	230 (32.0)	125 (38.7)	
Education level				
Secondary or under	165 (26.4)	249 (34.7)	135 (41.8)	**<.001**
High or equal	303 (48.5)	273 (38.0)	109 (33.7)	
Bachelor or above	157 (25.1)	196 (27.3)	79 (24.5)	
Household monthly income (RMB: yuan)				
≤4,999	336 (53.8)	344 (47.9)	155 (48.0)	**<.001**
5000–9999	210 (33.6)	216 (30.1)	98 (30.3)	
≥10,000	79 (12.6)	158 (22.0)	70 (21.7)	
Dietary intakes				
Cereals and potatoes < 250 g/day	255 (40.8)	283 (39.4)	116 (35.9)	.342
Vegetables < 300 g/day	331 (53.0)	346 (48.2)	147(45.5)	.063
Fruits < 200 g/day	402 (64.3)	490 (68.2)	238 (73.7)	**.013**
Meat and poultry < 40 g/day	305 (48.8)	232 (32.3)	83 (25.7)	**<.001**
Aquatic product < 40 g/day	508 (81.3)	548 (76.3)	216 (66.9)	**<.001**
Eggs < 40 g/day	285 (45.6)	320 (44.6)	150 (46.4)	.841
Milk and dairy products < 300 g/day	595 (95.2)	668 (93.0)	309 (95.7)	.121
Soybean and nuts < 25 g/day	244 (39.0)	278 (38.7)	124 (38.4)	.980
Use of vitamin D supplementation	38 (6.1)	66 (9.2)	37 (11.5)	**.012**
Regular drinking	144 (23.0)	212 (29.5)	106 (32.8)	**.002**
Current smoking status				
Smoker	66 (10.6)	95 (13.2)	54 (16.7)	**.026**
Never or quit smoking	559 (89.4)	623 (86.8)	269 (83.3)	
Physical activity				
Low	231 (37.0)	216 (30.1)	71 (22.0)	**<.001**
Medium	196 (31.4)	211 (29.4)	107 (33.1)	
High	153 (24.5)	253 (35.2)	126 (39.0)	
Unable to determine	45 (7.2)	38 (5.3)	19 (5.9)	
Latitude of survey points				
≤37°	321 (51.4)	584 (81.3)	285 (88.2)	**<.001**
>37°	304 (48.6)	134 (18.7)	38 (11.8)	
Obesity measurements (median (IQR))				
BMI	23.7 (4.8)	23.4 (4.6)	24.0 (4.6)	.107
ABSI	0.078 (0.007)	0.079 (0.007)	0.079 (0.007)	**.002**
BRI	3.55 (1.9)	3.61 (1.8)	3.69 (1.6)	.721

Note

Chi‐squared test was used for categorical variables and Kruskal–Wallis test for continuous non‐normal variables. Bold text represents a statistically significant difference (*p* <.05).

### 3.2 Multinomial logistic regressions for 25(OH)D status

After adjustment for possible influential factors (Table 2), no significant association was observed between BMI and 25(OH)D deficiency or insufficiency. Compared with Q1, participants in Q4 of ABSI and BRI were 2.334 and 2.215 times more likely to be in the 25(OH)D deficiency group. In terms of 25(OH)D insufficiency, only the ABSI was found to reach statistical significance. In sensitivity analyses conducted after multiple imputation, results were similar to the current analyses (Table S1).

**TABLE 2:** Association between BMI, ABSI, and BRI with serum 25(OH)D status, OR (95%CI)

Indices	Serum 25(OH)D			
	Deficiency		Insufficiency	
	Crude^a^	Adjusted^b^	Crude^a^	Adjusted^b^
BMI categories				
Under/normal weight	1.00 (Ref.)	1.00 (Ref.)	1.00 (Ref.)	1.00 (Ref.)
Overweight	0.826 (0.619–1.102)	1.068 (0.763–1.494)	0.759 (0.573–1.005)	0.843 (0.626–1.133)
Obesity	1.163 (0.739–1.830)	1.393 (0.828–2.343)	0.878 (0.557–1.386)	0.996 (0.618–1.607)
*p* for trend	.905	.268	.166	.573
ABSI categories^c^				
Q1	1.00 (Ref.)	1.00 (Ref.)	1.00 (Ref.)	1.00 (Ref.)
Q2	1.325 (0.901–1.948)	1.472 (0.946–2.291)	**1.757 (1.199–2.573)**	**1.894 (1.269–2.826)**
Q3	0.925 (0.646–1.325)	1.255 (0.809–1.946)	1.027 (0.716–1.473)	1.267 (0.850–1.887)
Q4	1.071 (0.733–1.567)	**2.334 (1.458–3.734)**	**1.676 (1.156–2.429)**	**2.372 (1.558–3.612)**
*p* for trend	.842	**.002**	.082	**.001**
BRI categories^c^				
Q1	1.00 (Ref.)	1.00 (Ref.)	1.00 (Ref.)	1.00 (Ref.)
Q2	1.202 (0.815–1.772)	1.479 (0.945–2.316)	1.204 (0.824–1.758)	1.400 (0.937–2.090)
Q3	0.739 (0.510–1.069)	0.977 (0.619–1.541)	0.785 (0.549–1.123)	0.931 (0.624–1.391)
Q4	1.285 (0.873–1.890)	**2.215 (1.365–3.594)**	1.139 (0.779–1.666)	1.459 (0.945–2.252)
*p* for trend	.702	**.010**	.890	.297

^a^ Unadjusted.

^b^ Adjusted for sex, age, education level, monthly household income, dietary intakes (vegetables, fruits, meat and poultry, aquatic product, and use of vitamin D supplements), current smoking and drinking status, physical activity, and latitude of survey points.

^c^ Sex‐specific quartiles. Reference category was the group of 25(OH)D sufficiency. Bold text represents a statistically significant difference (*p* <.05).

**FIGURE 1:**
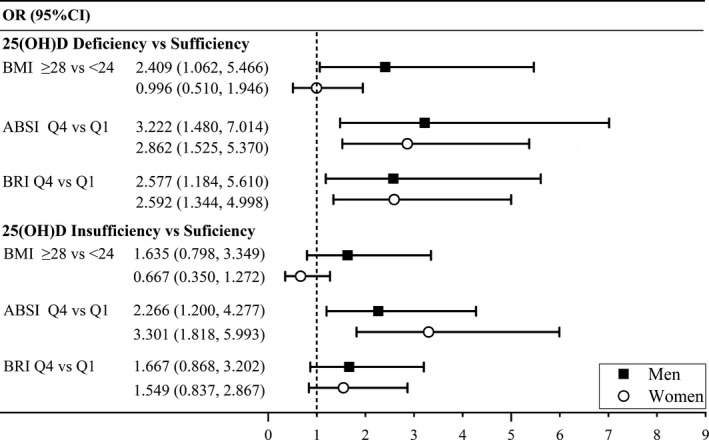
OR (95% CI) for BMI, ABSI, and BRI with 25(OH)D deficiency and insufficiency by sex. Results were derived from multinomial logistic regression analyses. All analyses were adjusted for age, education level, monthly household income, dietary intakes (vegetables, fruits, meat and poultry, aquatic product, and use of vitamin D supplements), current smoking and drinking status, physical activity, and latitude of survey points

Further analysis stratified by sex is shown in Figure 1. Among men, a significantly increased prevalence of 25(OH)D deficiency was observed for all three indices. Among women, a significant association with 25(OH)D deficiency was also found for ABSI and BRI, but not for BMI. When it comes to 25(OH)D insufficiency, the results for men and women were both in line with the entire study population. No significant correlation was observed except for ABSI.

### 3.3 Multivariate ROC analyses for 25(OH)D

**FIGURE 2:**
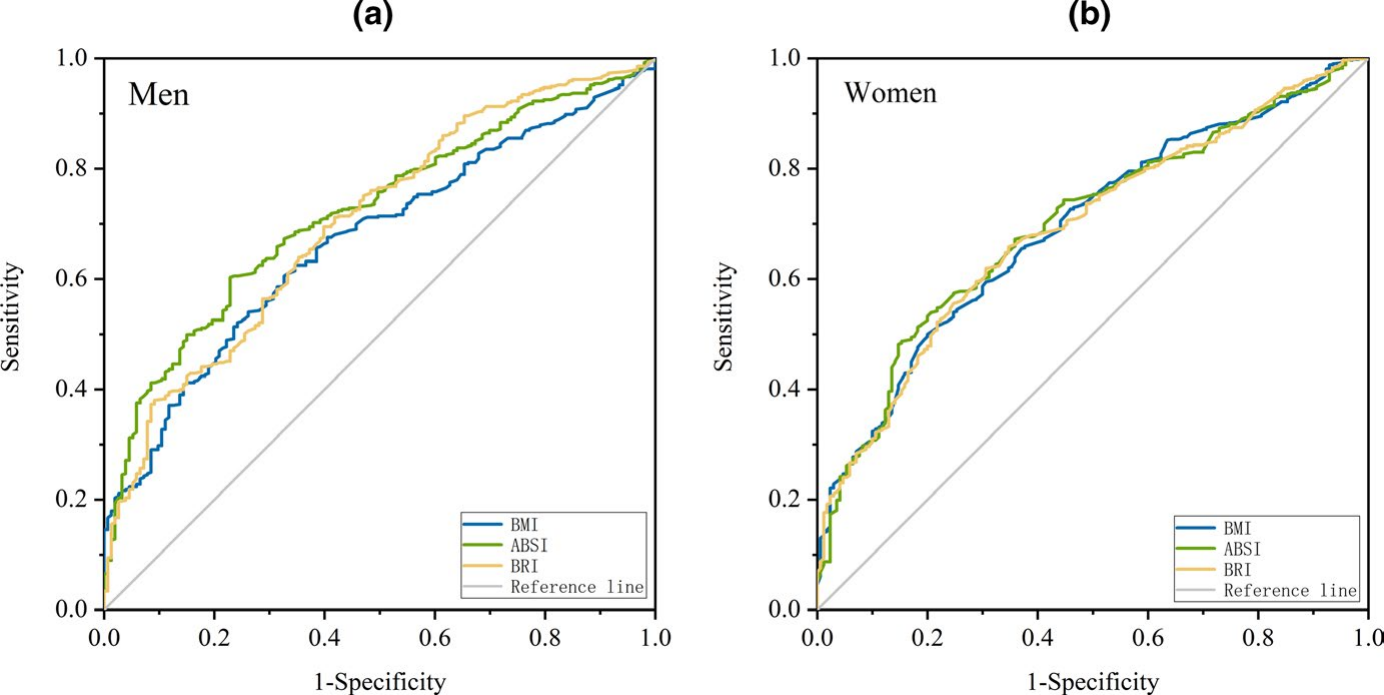
ROCs of BMI, ABSI, and BRI to identify low‐level 25(OH)D by sex. (a) Men; (b) women. ROCs were based on binary logistic regression adjusted for age, education level, monthly household income, dietary intakes (vegetables, fruits, meat and poultry, aquatic product, and use of vitamin D supplements), current smoking and drinking status, physical activity, and latitude of survey points. Areas for the curves are shown in Table 3

To predict a lower level of serum 25(OH)D (combining 25(OH)D deficiency and insufficiency into one group), ROCs of each anthropometric index were established using the predicted values based on binary logistic regressions, after adjusting for a series of possible influential factors (Figure 2). Overall, models with ABSI showed the largest AUCs among the three indices, while no significant difference was found. When stratified by sex, the AUCs of ABSI were significantly larger than that of BMI in men. However, no significant difference was observed in women (Table 3).

**TABLE 3:** AUCs of BMI, ABSI, and BRI for the presence of low‐level 25(OH)D in men and women

Indices	All		Men		Women	
	AUC	95%CI	AUC	95%CI	AUC	95%CI
BMI	0.716^a^	0.687–0.746	0.695^a^	0.649–0.741	0.722^a^	0.682–0.761
ABSI	0.725^a^	0.695–0.755	0.731^b^	0.687–0.774	0.723^a^	0.683–0.762
BRI	0.720^a^	0.690–0.750	0.715^ab^	0.668–0.761	0.720^a^	0.681–0.760

Note

Receiver operating characteristic curves were based on binary logistic regression. Delong test was used to compare the AUCs. Different letters in same column indicate significant differences between groups (*p* <.05).

## 4 DISCUSSION

Our study revealed that vitamin D deficiency and insufficiency were severe in Chinese adults, and the frequency of milk and dairy product or vitamin D supplement intake was also low. Despite the existence of gender heterogeneity, lower vitamin D levels may be one of the potential adverse consequences of obesity, supported by the significant associations between BMI, ABSI, and BRI with 25(OH)D found in this cross‐sectional study. Among these three indices, the ABSI showed a superior predictive ability for identifying low‐level serum 25(OH)D.

Using a method of radio‐labeling, the correlation between vitamin D and obesity was first reported in rats as early as in 1971, which suggested that vitamin D3 might be primarily stored in the adipose tissue (Rosenstreich et al., 1971). Since then, extensive research work has been carried out in different populations, of which BMI is the most commonly used anthropometric index to define obesity (Pourshahidi, 2015; Rafiq & Jeppesen, 2018; Saneei et al., 2013). Related systematic reviews and meta‐analyses were first published by Saneei et al., 2013, and a significant but weak association was reported, but not for women living in developing countries (Saneei et al., 2013), while in some other studies, the relation was stronger in women or did not exist (Bolland et al., 2007; Vanlint et al., 2011; Zhu et al., 2019). The discrepancies in published studies might be partly due to different study populations (developmental status, culture, lifestyle, diet, etc.), BMI groups, detections and definitions of the 25(OH)D status, residual confounders, and so on.

Two novel indices, ABSI and BRI, that took body shape into account were also applied in recent studies and have been implicated in a series of chronic diseases, including diabetes, cardiometabolic abnormalities (CMA), metabolic syndrome, and hyperuricemia (Calton et al., 2015; Ji et al., 2018; Kuk et al., 2005; Pradhan, 2014; Slagter et al., 2017). However, to the best of our knowledge, only two articles published at the present are related to serum vitamin D. Sousa‐Santos, A.R., et al. were the first to report that higher BRI and ABSI were associated with lower serum 25(OH)D (Q1) in older adults in Portugal (Sousa‐Santos et al., 2018). Another study conducted in central southern China by Zhu X‐L et al. founded that lower 25(OH)D (<30 ng/ml) was linked with increases odds of the highest quartile group of ABSI and BRI only in men (Zhu et al., 2019).

In line with Sousa‐Santos, A.R. and Zhu X‐L et al.’s results (Sousa‐Santos et al., 2018; Zhu et al., 2019), we did find strong relationships between obesity‐related indices and 25(OH)D: Compared with Q1, Q4 of ABSI and BRI had a significantly higher risk of 25(OH)D deficiency in both men and women. When 25(OH)D insufficiency was used as outcomes, the significant association was observed only with ABSI. However, the serum 25(OH)D deficiency was related to BMI only in men in our study.

These results might be a potentially evidence to understand how differences between indices may relate to 25(OH)D levels. As a traditional index, BMI was a good predictor of TBF, but its ability to distinguish fat from muscle and reflect fat distribution was limited (Aly et al., 2016). On the other hand, growing studies indicated that both ABSI and BRI showed a better ability to identify AAT or VAT (Krakauer & Krakauer, 2012; Thomas et al., 2013). In addition, recent studies revealed that the gender difference in body fat distribution should also be considered. Computed tomography measurements showed that, for a given TBF, men tended to have significantly more VAT or hepatic fat than women (Bawadi et al., 2019; Gomez‐Peralta et al., 2018). For a given WC, men had less total abdominal adipose tissue (TAAT) or aSAT than did women (Tian et al., 2016). A further study by Rafiq et al. (2019) indicated a gender difference whereby VAT and TBF were found to be associated with 25(OH)D in women, whereas VAT and hepatic fat showed the same association in men. Among all the fat deposits, VAT showed the strongest associations, which might be due to its metabolic activity of secreting proinflammatory adipokines (Rafiq et al., 2019; Zhang et al., 2016). These underlying mechanisms may at least in part explain the lack of association between BMI and 25(OH)D in women in our study.

In addition, we applied multivariate ROC analyses to compare the ability of these three anthropometric indices of screening 25(OH)D deficiency or insufficiency for the first time, and the models with ABSI showed the highest value of AUC, even though they were not very high. Gender stratification revealed that the difference was more pronounced in men than in women. Multivariate ROC analyses of these indices were also used previously for the prediction of hyperuricemia, while ABSI showed the lowest value (around 0.580) in both men and women (Calton et al., 2015). However, comparability between these studies is limited as the outcomes vary. Further studies are still needed to make up for the paucity of serum vitamin D studies.

Although our study provides interesting results, it was not possible to verify the temporal sequence between exposure and outcome due to the cross‐sectional study design, so a causal inference cannot be established. Considering the evidence of several interventional studies that higher BMI did negatively influence the response to supplemental vitamin D (Pourshahidi, 2015), in this study we tended to assume that vitamin D deficiency is a kind of malnutrition within morbidly obese population, while some researchers hypothesized that it is the cause of obesity (Foss, 2009; Mansouri et al., 2019; Mathieu et al., 2018). Despite the growing body of evidence, no prospective or randomized controlled studies are currently available that address the regulation of ABSI or BRI on serum 25(OH)D. Additionally, the strength of association varied among previous studies. There remains much need for further elucidation.

Several other limitations should also be mentioned. Although we have adjusted for multiple confounding factors, daily dietary vitamin D intakes were not been obtained due to data limitations. Nor did we have baseline data on indoor/outdoor work or the season of sampling, which could be related to sun exposure and thereby levels of vitamin D. Nonetheless, it must be kept in mind that anthropometric indices are not true measures of body distribution. In addition to the fat distribution mentioned earlier, recent study has suggested that vitamin D levels are associated with muscle mass (Hassan‐Smith et al., 2017), which indicated that the type of obesity (sarcopenic/nonsarcopenic) potentially confounded study results. Future studies combined with imaging diagnostic methods, such as magnetic resonance imaging or computed tomography, will contribute to clarifying the associations and promoting the clinical application of these indices.

## 5 CONCLUSION

A positive association between obesity and lower 25(OH)D serum concentration was found among Chinese adults. Besides BMI, novel obesity‐related indicator, ABSI and BRI were associated with lower serum 25(OH)D to some extent, and further studies are needed to clarify their potential to be used as screening tools in clinical practice.

## CONFLICT OF INTEREST

The authors declare that they have no conflict of interest.

## ETHICAL STATEMENT

The study conforms to the Declaration of Helsinki and does not embrace any human or animal testing. The study's protocols and procedures were ethically reviewed and approved by the Medical Ethics Research Board of Peking University (No. IRB00001052‐15059). Written informed consent from every participant had been obtained and documented.

## Supporting information

Table S1

## References

[fsn32201-bib-0001] Aly, Y. E. , Abdou, A. S. , Rashad, M. M. , & Nassef, M. M. (2016). Effect of exercise on serum vitamin D and tissue vitamin D receptors in experimentally induced type 2 Diabetes Mellitus. Journal of Advanced Research, 7(5), 671–679. 10.1016/j.jare.2016.07.001 27504197 PMC4969241

[fsn32201-bib-0002] Barrea, L. , Muscogiuri, G. , Annunziata, G. , Laudisio, D. , de Alteriis, G. , Tenore, G. C. , Colao, A. , & Savastano, S. (2019). A New Light on Vitamin D in Obesity: A Novel Association with Trimethylamine‐N‐Oxide (TMAO). Nutrients, 11(6), 1310. 10.3390/nu11061310 PMC6627576 31185686

[fsn32201-bib-0003] Bawadi, H. , Abouwatfa, M. , Alsaeed, S. , Kerkadi, A. , & Shi, Z. (2019). Body Shape Index Is a Stronger Predictor of Diabetes. Nutrients, 11(5), 1018. 10.3390/nu11051018 PMC6566958 31067681

[fsn32201-bib-0004] Bischoff‐Ferrari, H. A. (2012). Relevance of vitamin D in muscle health. Reviews in Endocrine & Metabolic Disorders, 13(1), 71–77. 10.1007/s11154-011-9200-6 22020957

[fsn32201-bib-0005] Bolland, M. J. , Grey, A. B. , Ames, R. W. , Horne, A. M. , Mason, B. H. , Wattie, D. J. , Gamble, G. D. , Bouillon, R. , & Reid, I. R. (2007). Age‐, gender‐, and weight‐related effects on levels of 25‐hydroxyvitamin D are not mediated by vitamin D binding protein. Clinical Endocrinology, 67(2), 259–264. 10.1111/j.1365-2265.2007.02873.x 17547688

[fsn32201-bib-0006] Buuren, S. V. , & Groothuis‐Oudshoorn, K. (2011). Mice: Multivariate Imputation by Chained Equations in R. Journal of Statistical Software, 45(3), 67. 10.18637/jss.v045.i03

[fsn32201-bib-0007] Calton, E. K. , Keane, K. N. , Newsholme, P. , & Soares, M. J. (2015). The Impact of Vitamin D Levels on Inflammatory Status: A Systematic Review of Immune Cell Studies. PLoS One, 10(11), e0141770. 10.1371/journal.pone.0141770 26528817 PMC4631349

[fsn32201-bib-0008] Cheng, S. , Massaro, J. M. , Fox, C. S. , Larson, M. G. , Keyes, M. J. , McCabe, E. L. , Robins, S. J. , O'Donnell, C. J. , Hoffmann, U. , Jacques, P. F. , Booth, S. L. , Vasan, R. S. , Wolf, M. , & Wang, T. J. (2010). Adiposity, cardiometabolic risk, and vitamin D status: The Framingham Heart Study. Diabetes, 59(1), 242–248. 10.2337/db09-1011 19833894 PMC2797928

[fsn32201-bib-0009] Chinese Nutrition Association (2016). Dietary Guidelines for Chinese Residents (2016th ed). People's Medical Publishing House.

[fsn32201-bib-0010] Drafting committee of Chinese consensus on overweight/obesity medical nutrition therapy (2016). Chinese consensus on overweight/obesity medical nutrition theray. Chinese Journal of Diabetes Mellitus, 8(9), 525–540. 10.3760/cma.j.issn.1674-5809.2016.09.004

[fsn32201-bib-0011] Drincic, A. T. , Armas, L. A. , Van Diest, E. E. , & Heaney, R. P. (2012). Volumetric dilution, rather than sequestration best explains the low vitamin D status of obesity. Obesity (Silver Spring), 20(7), 1444–1448. 10.1038/oby.2011.404 22262154

[fsn32201-bib-0012] Foss, Y. J. (2009). Vitamin D deficiency is the cause of common obesity. Medical Hypotheses, 72(3), 314–321. 10.1016/j.mehy.2008.10.005 19054627

[fsn32201-bib-0013] Gomez‐Peralta, F. , Abreu, C. , Cruz‐Bravo, M. , Alcarria, E. , Gutierrez‐Buey, G. , Krakauer, N. Y. , & Krakauer, J. C. (2018). Relationship between "a body shape index (ABSI)" and body composition in obese patients with type 2 diabetes. Diabetology & Metabolic Syndrome, 10, 21. 10.1186/s13098-018-0323-8 29568333 PMC5859756

[fsn32201-bib-0014] Hassan‐Smith, Z. K. , Jenkinson, C. , Smith, D. J. , Hernandez, I. , Morgan, S. A. , Crabtree, N. J. , Gittoes, N. J. , Keevil, B. G. , Stewart, P. M. , & Hewison, M. (2017). 25‐hydroxyvitamin D3 and 1,25‐dihydroxyvitamin D3 exert distinct effects on human skeletal muscle function and gene expression. PLoS One, 12(2), e0170665. 10.1371/journal.pone.0170665 28199350 PMC5310904

[fsn32201-bib-0015] Holick, M. F. (2005). The influence of vitamin D on bone health across the life cycle. Journal of Nutrition, 135(11), 2726s–2727s. 10.1093/jn/135.11.2726S 16251638

[fsn32201-bib-0016] Holick, M. F. (2007). Vitamin D deficiency. New England Journal of Medicine, 357(3), 266–281. 10.1056/NEJMra070553 17634462

[fsn32201-bib-0017] Holick, M. F. , Binkley, N. C. , Bischoff‐Ferrari, H. A. , Gordon, C. M. , Hanley, D. A. , Heaney, R. P. , Murad, M. H. , & Weaver, C. M. (2011). Evaluation, treatment, and prevention of vitamin D deficiency: An Endocrine Society clinical practice guideline. Journal of Clinical Endocrinology and Metabolism, 96(7), 1911–1930. 10.1210/jc.2011-0385 21646368

[fsn32201-bib-0018] Holick, M. F. , & Chen, T. C. (2008). Vitamin D deficiency: A worldwide problem with health consequences. American Journal of Clinical Nutrition, 87(4), 1080s–1086s. 10.1093/ajcn/87.4.1080S 18400738

[fsn32201-bib-0019] IPAQ group (2005). Guidelines for data processing and analysis of the International Physical Activity Questionnaire (IPAQ). Retrieved from https://sites.google.com/site/theipaq/scoring‐protocol

[fsn32201-bib-0020] James, W. P. T. (2018). Obesity: A Global Public Health Challenge. Clinical Chemistry, 64(1), 24–29. 10.1373/clinchem.2017.273052 29295834

[fsn32201-bib-0021] Ji, M. , Zhang, S. , & An, R. (2018). Effectiveness of A Body Shape Index (ABSI) in predicting chronic diseases and mortality: A systematic review and meta‐analysis. Obesity Reviews, 19(5), 737–759. 10.1111/obr.12666 29349876

[fsn32201-bib-0022] Krakauer, N. Y. , & Krakauer, J. C. (2012). A new body shape index predicts mortality hazard independently of body mass index. PLoS One, 7(7), e39504. 10.1371/journal.pone.0039504 22815707 PMC3399847

[fsn32201-bib-0023] Kuk, J. L. , Lee, S. , Heymsfield, S. B. , & Ross, R. (2005). Waist circumference and abdominal adipose tissue distribution: Influence of age and sex. American Journal of Clinical Nutrition, 81(6), 1330–1334. 10.1093/ajcn/81.6.1330 15941883

[fsn32201-bib-0024] Liao, X. , Zhang, Z. , Zhang, H. , Zhu, H. , Zhou, J. , Huang, Q. , Liu, Z. (2014). Application Guideline for Vitamin D and Bone Health in Adult Chinese (2014 Standard Edition). Chinese Journal of Osteoporosis, 20(9), 1011–1030.

[fsn32201-bib-0025] Looker, A. C. (2007). Do body fat and exercise modulate vitamin D status? Nutrition Reviews, 65(8 Pt 2), S124–126. 10.1301/nr.2007.aug.s124-s126 17867388

[fsn32201-bib-0026] Lu, L. , Yu, Z. , Pan, A. , Hu, F. B. , Franco, O. H. , Li, H. , Li, X. , Yang, X. , Chen, Y. , & Lin, X. (2009). Plasma 25‐hydroxyvitamin D concentration and metabolic syndrome among middle‐aged and elderly Chinese individuals. Diabetes Care, 32(7), 1278–1283. 10.2337/dc09-0209 19366976 PMC2699709

[fsn32201-bib-0027] Malmberg, P. , Karlsson, T. , Svensson, H. , Lönn, M. , Carlsson, N.‐G. , Sandberg, A.‐S. , Jennische, E. , Osmancevic, A. , & Holmäng, A. (2014). A new approach to measuring vitamin D in human adipose tissue using time‐of‐flight secondary ion mass spectrometry: A pilot study. Journal of Photochemistry and Photobiology B: Biology, 138, 295–301. 10.1016/j.jphotobiol.2014.06.008 25010290

[fsn32201-bib-0028] Mansouri, M. , Miri, A. , Varmaghani, M. , Abbasi, R. , Taha, P. , Ramezani, S. , Rahmani, E. , Armaghan, R. , & Sadeghi, O. (2019). Vitamin D deficiency in relation to general and abdominal obesity among high educated adults. Eating and Weight Disorders, 24(1), 83–90. 10.1007/s40519-018-0511-4 29856006

[fsn32201-bib-0029] Martini, L. A. , & Wood, R. J. (2006). Vitamin D status and the metabolic syndrome. Nutrition Reviews, 64(11), 479–486. 10.1111/j.1753-4887.2006.tb00180.x 17131943

[fsn32201-bib-0030] Mathieu, S.‐V. , Fischer, K. , Dawson‐Hughes, B. , Freystaetter, G. , Beuschlein, F. , Schietzel, S. , Egli, A. , & Bischoff‐Ferrari, H. (2018). Association between 25‐Hydroxyvitamin D Status and Components of Body Composition and Glucose Metabolism in Older Men and Women. Nutrients, 10(12), 10.3390/nu10121826 PMC6315833 30477276

[fsn32201-bib-0031] Pereira‐Santos, M. , Costa, P. R. , Assis, A. M. , Santos, C. A. , & Santos, D. B. (2015). Obesity and vitamin D deficiency: A systematic review and meta‐analysis. Obesity Reviews, 16(4), 341–349. 10.1111/obr.12239 25688659

[fsn32201-bib-0032] Pourshahidi, L. K. (2015). Vitamin D and obesity: Current perspectives and future directions. The Proceedings of the Nutrition Society, 74(2), 115–124. 10.1017/s0029665114001578 25359323

[fsn32201-bib-0033] Pradhan, A. D. (2014). Sex differences in the metabolic syndrome: Implications for cardiovascular health in women. Clinical Chemistry, 60(1), 44–52. 10.1373/clinchem.2013.202549 24255079

[fsn32201-bib-0034] Rafiq, R. , Walschot, F. , Lips, P. , Lamb, H. J. , de Roos, A. , Rosendaal, F. R. , Heijer, M. D. , de Jongh, R. T. , & de Mutsert, R. (2019). Associations of different body fat deposits with serum 25‐hydroxyvitamin D concentrations. Clinical Nutrition, 38(6), 2851–2857. 10.1016/j.clnu.2018.12.018 30635144

[fsn32201-bib-0035] Rafiq, S. , & Jeppesen, P. B. (2018). Body Mass Index, Vitamin D, and Type 2 Diabetes: A Systematic Review and Meta‐Analysis. Nutrients, 10(9), 1182. 10.3390/nu10091182 PMC6164132 30154381

[fsn32201-bib-0036] Rosenstreich, S. J. , Rich, C. , & Volwiler, W. (1971). Deposition in and release of vitamin D3 from body fat: evidence for a storage site in the rat. Journal of Clinical Investigation, 50(3), 679–687. 10.1172/JCI106538 PMC291976 4322721

[fsn32201-bib-0037] Saneei, P. , Salehi‐Abargouei, A. , & Esmaillzadeh, A. (2013). Serum 25‐hydroxy vitamin D levels in relation to body mass index: A systematic review and meta‐analysis. Obesity Reviews, 14(5), 393–404. 10.1111/obr.12016 23331724

[fsn32201-bib-0038] Slagter, S. N. , van Waateringe, R. P. , van Beek, A. P. , van der Klauw, M. M. , Wolffenbuttel, B. H. R. , & van Vliet‐Ostaptchouk, J. V. (2017). Sex, BMI and age differences in metabolic syndrome: The Dutch Lifelines Cohort Study. Endocrine Connections, 6(4), 278–288. 10.1530/ec-17-0011 28420718 PMC5457493

[fsn32201-bib-0039] Sousa‐Santos, A. R. , Afonso, C. , Santos, A. , Borges, N. , Moreira, P. , Padrão, P. , Fonseca, I. , & Amaral, T. F. (2018). The association between 25(OH)D levels, frailty status and obesity indices in older adults. PLoS One, 13(8), e0198650. 10.1371/journal.pone.0198650 30153256 PMC6112621

[fsn32201-bib-0040] Thomas, D. M. , Bredlau, C. , Bosy‐Westphal, A. , Mueller, M. , Shen, W. , Gallagher, D. , Maeda, Y. , McDougall, A. , Peterson, C. M. , Ravussin, E. , & Heymsfield, S. B. (2013). Relationships between body roundness with body fat and visceral adipose tissue emerging from a new geometrical model. Obesity (Silver Spring), 21(11), 2264–2271. 10.1002/oby.20408 23519954 PMC3692604

[fsn32201-bib-0041] Tian, S. , Zhang, X. , Xu, Y. , & Dong, H. (2016). Feasibility of body roundness index for identifying a clustering of cardiometabolic abnormalities compared to BMI, waist circumference and other anthropometric indices: The China Health and Nutrition Survey, 2008 to 2009. Medicine, 95(34), e4642. 10.1097/MD.0000000000004642 27559964 PMC5400331

[fsn32201-bib-0042] Vanlint, S. J. , Morris, H. A. , Newbury, J. W. , & Crockett, A. J. (2011). Vitamin D insufficiency in Aboriginal Australians. Medical Journal of Australia, 194(3), 131–134. 10.5694/j.1326-5377.2011.tb04195.x 21299487

[fsn32201-bib-0043] Wood, G. C. , Chu, X. , Manney, C. , Strodel, W. , Petrick, A. , Gabrielsen, J. , Seiler, J. , Carey, D. , Argyropoulos, G. , Benotti, P. , Still, C. D. , & Gerhard, G. S. (2012). An electronic health record‐enabled obesity database. BMC Medical Informatics and Decision Making, 12, 45. 10.1186/1472-6947-12-45 22640398 PMC3508953

[fsn32201-bib-0044] Zhang, N. , Chang, Y. , Guo, X. , Chen, Y. , Ye, N. , & Sun, Y. (2016). A Body Shape Index and Body Roundness Index: Two new body indices for detecting association between obesity and hyperuricemia in rural area of China. European Journal of Internal Medicine, 29, 32–36. 10.1016/j.ejim.2016.01.019 26895753

[fsn32201-bib-0045] Zhao, A. I. , Szeto, I. , Wang, Y. , Li, C. E. , Pan, M. , Li, T. , Wang, P. , & Zhang, Y. (2017). Knowledge, Attitude, and Practice (KAP) of Dairy Products in Chinese Urban Population and the Effects on Dairy Intake Quality. Nutrients, 9(7), 668. 10.3390/nu9070668

[fsn32201-bib-0046] Zhu, X.‐L. , Chen, Z.‐H. , Li, Y. , Yang, P.‐T. , Liu, L. , Wu, L.‐X. , & Wang, Y.‐Q. (2019). Associations of vitamin D with novel and traditional anthropometric indices according to age and sex: a cross‐sectional study in central southern China. Eating and Weight Disorders ‐ Studies on Anorexia, Bulimia and Obesity, 25(6), 1651–1661. 10.1007/s40519-019-00803-8 31728924

