# Supplementary material for: Association of serum 25‐hydroxy vitamin D with obesity‐related indices in Chinese adults: A cross‐sectional study
Source: Food Sci Nutr. 2021 Feb 26;9(4):2260–8. doi: 10.1002/fsn3.2201 (PMC8020955; doi:10.1002/fsn3.2201)
Supplement: Supplementary file 1 — Table S1 [file FSN3-9-2260-s001.docx]

**Table S1** Association between BMI, ABSI and BRI with serum 25(OH)D status in sensitivity analyses, OR (95%CI)

| **Indices** | **Serum 25(OH)D** | | | | |
| --- | --- | --- | --- | --- | --- |
|  | **Deficiency** | |  | **Insufficiency** | |
|  | **Crude ^a^** | **Adjusted ^b^** |  | **Crude ^a^** | **Adjusted ^b^** |
| **BMI categories** |  |  |  |  |  |
| Under/normal weight | 1.00 (Ref.) | 1.00 (Ref.) |  | 1.00 (Ref.) | 1.00 (Ref.) |
| Over weight | 0.833 (0.626-1.107) | 1.096 (0.785-1.531) |  | **0.749 (0.567-0.990)** | 0.853 (0.635-1.147) |
| Obesity | 1.162 (0.739-1.829) | 1.419 (0.845-2.383) |  | 0.916 (0.582-1.441) | 1.032 (0.641-1.662) |
| *p* for trend | 0.834 | 0.220 |  | 0.149 | 0.678 |
| **ABSI categories** ^c^ |  |  |  |  |  |
| Q1 | 1.00 (Ref.) | 1.00 (Ref.) |  | 1.00 (Ref.) | 1.00 (Ref.) |
| Q2 | 1.304 (0.888-1.917) | 1.482 (0.953-2.303) |  | **1.710 (1.168-2.504)** | **1.865 (1.250-2.783)** |
| Q3 | 0.903 (0.631-1.293) | 1.222 (0.789-1.893) |  | 1.009 (0.706-1.443) | 1.262 (0.849-1.877) |
| Q4 | 1.054 (0.723-1.536) | **2.348 (1.470-3.751)** |  | **1.563 (1.081-2.259)** | **2.336 (1.535-3.554)** |
| *p* for trend | 0.728 | **0.002** |  | 0.177 | **0.001** |
| **BRI categories** ^c^ |  |  |  |  |  |
| Q1 | 1.00 (Ref.) | 1.00 (Ref.) |  | 1.00 (Ref.) | 1.00 (Ref.) |
| Q2 | 1.187 (0.808-1.745) | 1.479 (0.946-2.312) |  | 1.159 (0.796-1.689) | 1.378 (0.924-2.055) |
| Q3 | 0.732 (0.506-1.058) | 1.010 (0.641-1.593) |  | 0.760 (0.531-1.088) | 0.932 (0.624-1.392) |
| Q4 | 1.271 (0.868-1.862) | **2.268 (1.399-3.675)** |  | 1.096 (0.753-1.597) | 1.479 (0.959-2.281) |
| *p* for trend | 0.791 | **0.006** |  | 0.698 | 0.258 |

^a^ Unadjusted. ^b^ Adjusted for sex, age, education level, monthly household income, dietary intakes (vegetables, fruits, meat and poultry, aquatic product, and use of vitamin D supplements), current smoking and drinking status, physical activity, and latitude of survey points. ^c^ Sex-specific quartiles. Reference category was the group of 25(OH)D sufficiency. Bold text represents a statistically significant difference (*p*＜0.05).
